# Corrigendum to “PAX3: A Molecule with Oncogenic or Tumor Suppressor Function Is Involved in Cancer”

**DOI:** 10.1155/2019/3401505

**Published:** 2019-12-09

**Authors:** Ashok Arasu, Sengottuvelan Murugan, Musthafa Mohamed Essa, Thirunavukkarasu Velusamy, Gilles J. Guillemin

**Affiliations:** ^1^Department of Biotechnology, School of Biotechnology and Genetic Engineering, Bharathiar University, Coimbatore 641046, Tamil Nadu, India; ^2^Endocrine Research Facility, Department of Animal Science, Rutgers University, New Brunswick-08901, NJ, USA; ^3^Department of Food Science and Nutrition, CAMS, Sultan Qaboos University, Muscat, Oman; ^4^Neuroinflammation Group, Faculty of Medicine and Health Sciences, Deb Bailey MND Research Laboratory, Macquarie University, NSW, 2109, Australia

In the article titled “PAX3: A Molecule with Oncogenic or Tumor Suppressor Function Is Involved in Cancer” [1], Dr. Mathivanan Jothi was missed in the acknowledgements section. As a former mentor to Ashok Arasu contributing to his concepts, ideas and discussions on which review article is based, the acknowledgements section is updated to reflect this.
